# Overexpressed Palladin Rescues Enteropathogenic *E. coli* (EPEC) Pedestal Lengths in ArpC2 Depleted Cells

**DOI:** 10.1002/cm.21974

**Published:** 2024-12-18

**Authors:** Kaitlin M. Bruzzini, S. Tara Mann, Julian A. Guttman

**Affiliations:** ^1^ Department of Biological Sciences, Centre for Cell Biology, Development, and Disease Simon Fraser University Burnaby British Columbia Canada

**Keywords:** actin polymerization, Arp2/3 complex, EPEC, microbiology, Palladin, pedestals

## Abstract

Enteropathogenic *Escherichia coli* (EPEC) causes diarrheal disease. Once ingested, these extracellular pathogens attach to the intestinal epithelial cells of their host, collapse the localized microvilli, and generate actin‐rich structures within the host cells that are located beneath the attached bacteria, called “pedestals.” Palladin is an actin‐associated protein that cross‐links and stabilizes actin filaments. This protein also acts as a scaffolding protein for other actin‐binding proteins. Here, we examine the role of Palladin during EPEC infections and show that Palladin is co‐opted by EPEC. Depletion of Palladin resulted in shorter pedestals, and when Palladin containing mutations in either its actin‐ or VASP‐binding domains were overexpressed in cells, pedestals decreased in length. Importantly, we show that the overexpression of Palladin in *ArpC2* ^*−/−*^ (Arp2/3 complex–depleted) cells rescued pedestal length. Together, our results demonstrate that Palladin has the ability to rescue pedestal length during EPEC infections when the function of the Arp2/3 complex is diminished.

## 1 Introduction

Upon ingestion of enteropathogenic *Escherichia coli* (EPEC), infected individuals will present with a variety of gastrointestinal symptoms post‐exposure (Nataro and Kaper 1998). These extracellular bacteria are part of a group of microbes generally referred to as attaching and effacing (A/E) pathogens. Members of this group attach to the intestinal epithelial cells of their hosts and collapse (efface) the microvilli of those cells (Agin and Wolf 1997; Moon et al. 1983; Rothbaum et al. 1982; Staley, Jones, and Corley 1969). Additional morphological changes happen to the infected cells, the most prominently of which is the formation of actin‐rich “pedestal” structures at the top of the infected cells at sites of bacterial docking (Jerse et al. 1990; Knutton et al. 1987; Knutton, Lloyd, and McNeish 1987; Tzipori et al. 1985).

The generation of EPEC pedestals requires both host and bacterial sub‐cellular events to occur in a coordinated manner. The initial attachment of EPEC to the host intestinal epithelium is mediated by the bundle‐forming pilus (BFP) (Bieber et al. 1998; Girón, Ho, and Schoolnik 1991; Vuopio‐Varkila and Schoolnik 1991). In addition to BFP, surface‐expressed intimin plays a key role in mediating EPEC's adhesion to epithelial cells (Donnenberg and Kaper 1991; Jerse et al. 1990; Jerse and Kaper 1991). Upon attachment, the EPEC type III secretion system (T3SS) delivers the translocated intimin receptor (Tir) into the host cell (Jarvis et al. 1995; Kenny et al. 1997). This effector protein embeds itself into the host cell plasma membrane, where it interacts extracellularly with the bacterial surface protein intimin (De Grado et al. 1999; Deibel et al. 1998; Hartland et al. 1999; Kenny et al. 1997). The Tir–intimin interaction results in Tir clustering and stimulates its phosphorylation at Y474 (Campellone et al. 2004; Kenny 1999; Touzé et al. 2004). This phosphorylation recruits the host proteins Nck1 and Nck2, which in turn recruit and activateN‐WASp (Campellone et al. 2002, 2004; Gruenheid et al. 2001; Rohatgi et al. 2001). N‐WASp then recruits and activates the Arp2/3 (actin‐related protein) complex, leading to actin filament polymerization beneath the bacterial attachment site (Goosney, DeVinney, and Finlay 2001; Kalman et al. 1999; Lommel et al. 2001; Rohatgi et al. 2001). The abundant actin polymerization in that region creates a protrusion at the plasma membrane known as a “pedestal,” atop which EPEC sits (Jarvis et al. 1995; Kenny et al. 1997; Kenny and Finlay 1997; Rosenshine et al. 1992). While Nck recruitment via the phosphorylation of Y474 is an important mechanism by which EPEC triggers actin polymerization, an additional Nck‐independent mechanism exists. In the absence of Nck, phosphorylation of Tir at Y454 recruits N‐WASp, which then recruits the Arp2/3 complex for pedestal formation (Campellone and Leong 2005; Schüller et al. 2007).

The actin‐associated protein, Palladin, localizes to a variety of structures within cells, including stress fibres, cell–cell junctions, and membrane ruffles (Gateva et al. 2014; Goicoechea, Arneman, and Otey 2008; Parast and Otey 2000). A prominent feature of Palladin is its ability to bind to linear actin (Parast and Otey 2000) and cross‐link actin filaments (Dixon et al. 2008). Palladin also has the ability to form branched actin filament networks, as demonstrated through pure protein in vitro assays (Gurung et al. 2016), within comet tails formed by *Listeria monocytogenes* bacteria when the Arp2/3 complex is depleted, and during reconstitution experiments where pure Palladin protein is used in place of the Arp2/3 complex (Dhanda et al. 2018). Based on these features, we sought to determine whether Palladin plays a role during EPEC pedestal formation. We found that although Palladin localized within EPEC pedestals, it did not concentrate at regions of the pedestal where actin was most prominent. Upon Palladin depletion or overexpression with either a Palladin ΔVASP mutant or a Palladin construct mutated in its actin‐binding domain, pedestal height was significantly decreased. Most importantly, we found that Palladin overexpression could rescue pedestals to wild‐type lengths in ArpC2‐depleted cells.

## 2 Results

### 2.1 Palladin Localizes to the Base of EPEC Pedestals

**FIGURE 1:**
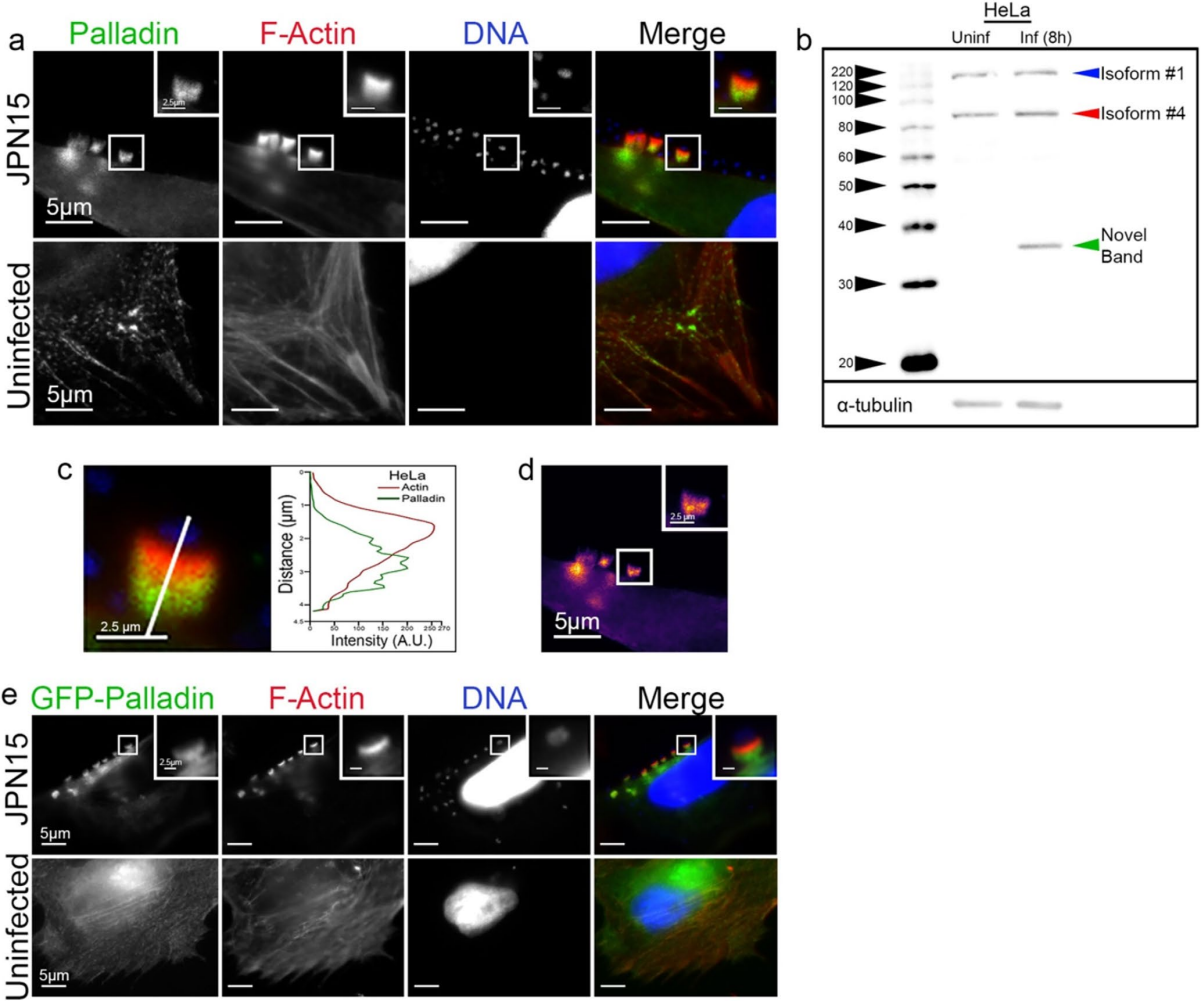
Localization of Palladin at EPEC pedestals. (a) HeLa cells were infected with EPEC (JPN15) for 8 h (h), fixed, and stained with Palladin‐targeting antibody (green), Alexa 594‐phalloidin (red) to visualize filamentous actin (F‐actin), and DAPI (blue) to visualize host cell DNA and bacteria. The insets are enlargements of boxed regions. Scale bars are 5 or 2.5 μm (inset). (b) Whole HeLa lysates from uninfected (Uninf) cells versus cells from 8 h EPEC infections (Inf) were probed for endogenous Palladin using mouse polyclonal anti‐palladin antibodies. Palladin isoform 4 is indicated by a solid red arrowhead at ~90 kDa. Additional bands show other palladin isoforms, as indicated by solid blue and green arrowheads. α‐Tubulin is shown as a loading control. (c) The distribution of the total F‐actin intensity (red), as well as the corresponding Palladin (green), was plotted across the EPEC pedestal (white line). Scale bar = 2.5 μm. (d) A heat map of the representative Palladin signal where light yellow indicates the highest signal intensity. Scale bars are 5 or 2.5 μm (inset). (e) Distribution of GFP‐Palladin in HeLa cells infected with JPN15 for 8 h. Cells were stained with Alexa 594‐phalloidin (red) to visualize F‐actin and DAPI (blue) to visualize host cell DNA and bacteria. Scale bars are 5 or 2.5 μm (inset).

To determine if Palladin was present at EPEC pedestals, we infected HeLa cells with the JPN15 strain of EPEC. These microbes are BFP negative and thus do not form microcolonies, allowing for greater ease in observing individual EPEC pedestals. Immunolocalization showed that endogenous Palladin was present within the pedestals and was concentrated beneath the area where actin staining was most intense (Figure 1). A similar localization pattern was found when a GFP‐Palladin construct was used during HeLa cell infections (Figure 1). Palladin expression levels did not noticeably change in infected cells. However, a low molecular weight band (~35 kDa) appeared in the infected samples (Figure 1).

### 2.2 Palladin Requires Phosphorylation of Tir to Localize Beneath EPEC

**FIGURE 2:**
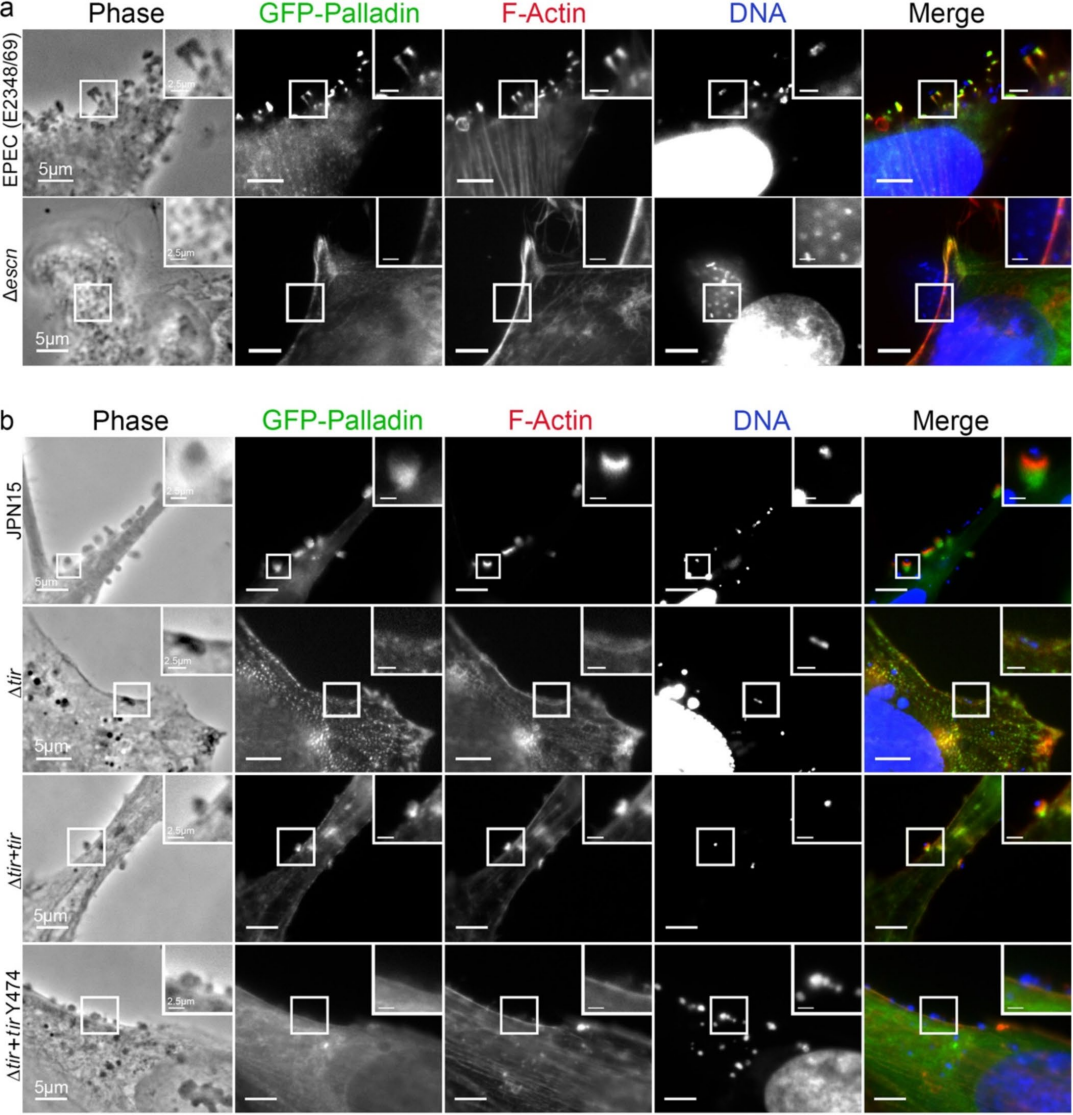
(a) Distribution of GFP‐Palladin at pedestals generated by EPEC (E2348/69) or EPEC (E2348/69) mutated in *escN* (Δ*escN*). Cells were stained with Alexa 594‐phalloidin (red) to visualize F‐actin and DAPI (blue) to visualize host cell DNA and bacteria. The insets are enlargements of boxed regions. Scale bars are 5 or 2.5 μm (inset). (b) Localization of GFP‐Palladin at sites of bacterial attachment during infections of HeLa cells with JPN15 and JPN15 effector mutants: 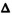 *tir*, ∆ *tir* + *tir*, and ∆ *tir* + *tir* Y474. Alexa 594‐phalloidin (red) to visualize F‐actin and DAPI (blue) to visualize host cell DNA and bacteria. The insets are enlargements of boxed regions. Scale bars are 5 or 2.5 μm (inset).

EPEC uses a T3SS to deliver effector proteins into their host cells to form pedestals (Jarvis et al. 1995). To determine whether EPEC attachment alone or pedestal formation was required to concentrate Palladin beneath EPEC when atop their host cells, we used a T3SS mutant strain of EPEC (EPEC Δ*escN*). When infected, Palladin did not localize to the sites of EPEC Δ*escN*, indicating that the attachment of the bacteria was not sufficient to trigger Palladin recruitment (Figure 2a). We further examined whether Tir or a key phosphorylation site on Tir (Y474) was needed for Palladin recruitment at sites of EPEC attachment, as these are required for pedestal formation. We found that in all cases, Palladin only concentrated beneath the bacterial attachment points when pedestals formed (when the Δ*tir* strain was complemented with *tir*, Δ*tir*+*tir*) (Figure 2b).

### 2.3 Knockdown (KD) of Palladin Shortens EPEC Pedestals

**FIGURE 3:**
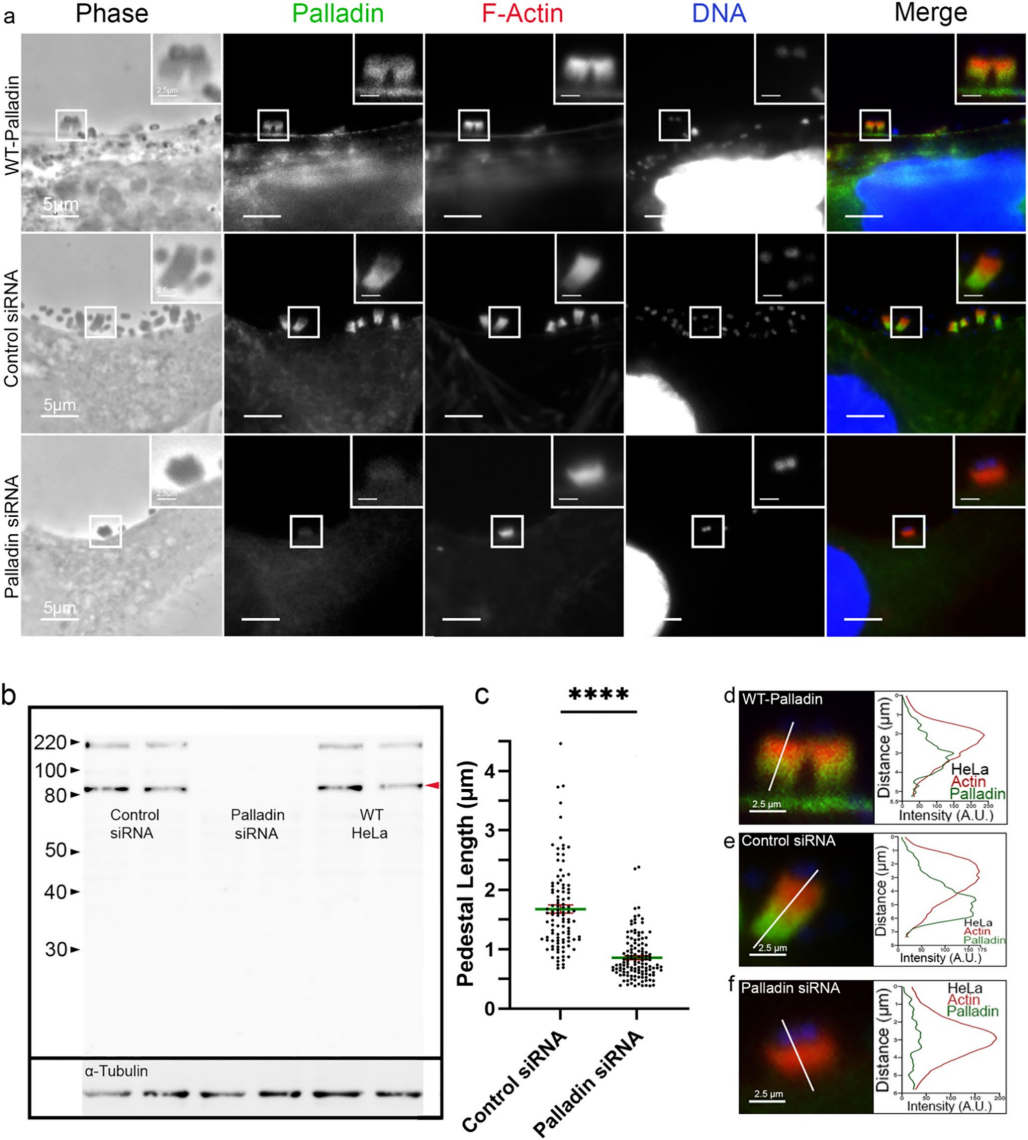
Effect of Palladin knockdowns on EPEC pedestal formation. (a) HeLa cells were treated with a smart pool of Palladin‐targeting siRNA, non‐targeting (control) siRNA, or were left untreated (WT HeLa). All samples were infected with EPEC for 8 h and then stained. Palladin polyclonal antibody (green), Alexa594‐phalloidin (red) to visualize F‐actin, and DAPI (blue) to visualize DNA and bacteria. Scale bars are 5 or 2.5 μm (inset). (b) Duplicate western blot lanes of HeLa cells treated with non‐targeting (control siRNA), Palladin‐targeted (Palladin siRNA) sequences, or untreated HeLa's are presented. Whole‐cell lysates were collected and probed for endogenous Palladin using a mouse polyclonal anti‐Palladin antibody. Palladin isoform 4 is located at 90 kDa (indicated by a solid red arrowhead); additional bands show other Palladin isoforms. The gel shown here was cropped from a larger gel and exposed for 3 min. α‐Tubulin was used as a loading control and exposed for 15 s. (c) Quantification of pedestal lengths from 3 separate experiments (where each experiment was run in triplicate) was obtained and plotted as a scatter plot (relative to control [±SEM]). The average pedestal length of the non‐targeting (control siRNA) pedestals was 1.676 μm (from 106 EPEC pedestals), while the average pedestal length of the KD‐Palladin pedestals (Palladin siRNA) was 0.8578 μm (from 126 EPEC pedestals). Asterisks indicate significant differences from siRNA control (*p* < 0.0001, unpaired parametric two‐tailed *t*‐tests [with Welch's correction]). The length of the pedestals was determined by looking at the F‐actin staining of the EPEC pedestal and measuring the top of the pedestal (just below the localization of the bug) to where the F‐actin‐associated with the pedestal meets the cellular membrane. (d–f) The white line was drawn through the EPEC pedestal to measure the distribution relative intensity of F‐actin intensity (red) and Palladin (green). Scale bar = 2.5 μm.

**FIGURE 4:**
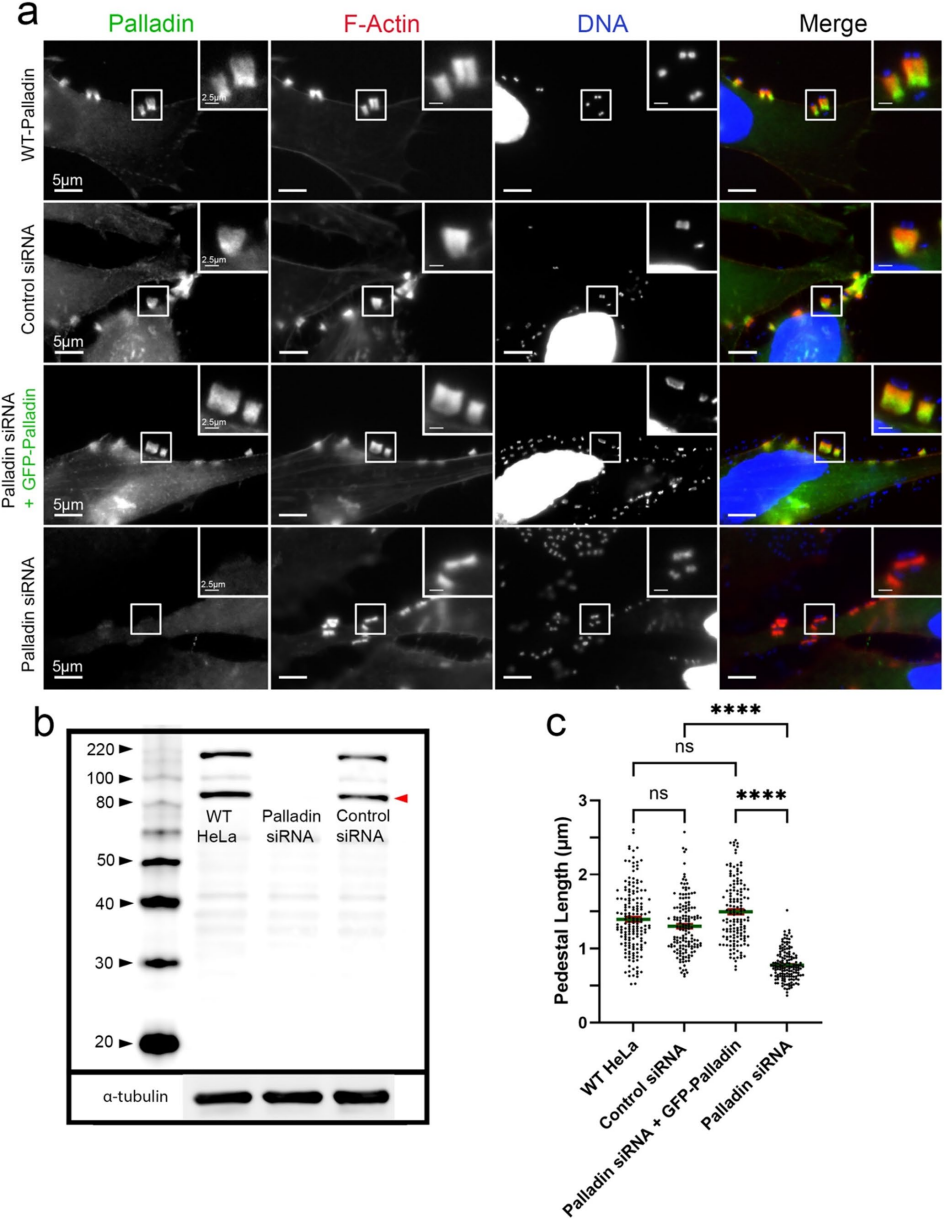
GFP‐Palladin restores pedestal length in Palladin knockdown cells. (a) HeLa cells were treated with a pool of 4 Palladin‐targeting siRNAs, non‐targeting (control) siRNAs, or were left untreated (WT HeLa). A population of Palladin siRNA‐treated cells was also transfected with GFP‐Palladin (Palladin siRNA + GFP‐Palladin). All samples were infected with EPEC for 8 h and then stained. Samples without GFP‐Palladin were treated with Palladin polyclonal antibody (green), Alexa594‐phalloidin (red) to visualize F‐actin, and DAPI (blue) to visualize DNA and bacteria. Samples with GFP‐Palladin were treated with Alexa594‐phalloidin (red) to visualize F‐actin and DAPI (blue) to visualize DNA and bacteria. Scale bars are 5 or 2.5 μm (inset). (b) Western blot of untreated HeLa cells, HeLa cells treated with Palladin‐targeted siRNA sequences (Palladin siRNA), or non‐targeting sequences (control siRNA). Whole‐cell lysates were collected and probed for endogenous Palladin. The Palladin isoform 4 is located at 90 kDa (indicated by a solid red arrowhead); additional bands show other Palladin isoforms. α‐Tubulin is shown as a loading control. (c) Quantification of pedestal lengths from three separate experiments (where each experiment was run in triplicate) was obtained and plotted as a scatter plot (relative to controls [±SEM]). The average pedestal length of the untreated WT‐HeLa control was 1.395 μm (from 166 EPEC pedestals), non‐targeting (control siRNA) pedestals was 1.302 μm (from 150 EPEC pedestals), while the average pedestal length of the KD‐Palladin + GFP‐Palladin pedestals was 1.496 μm (from 145 EPEC pedestals). The KD‐Palladin pedestals (Palladin siRNA) was 0.7753 μm (from 150 EPEC pedestals). One‐way analysis of variance (ANOVA) followed by Šidák multiple comparisons test was used to assess the significance of the EPEC pedestal length in WT‐HeLa compared with control siRNA (not significant [ns]), WT‐HeLa compared with Palladin siRNA + GFP‐Palladin (ns), control siRNA compared with Palladin siRNA (****p < 0.0001), and Palladin siRNA + GFP‐Palladin compared with Palladin siRNA (****p < 0.0001). Asterisks indicate significant differences.

To investigate the importance of Palladin during pedestal formation, we depleted Palladin in cultured cells using a smart pool of four Palladin small interfering RNAs (siRNAs). Concurrently, we treated cultured cells with a pool of four non‐targeting control siRNAs. The KDs were validated by Western blotting (Figure 3). The siRNA‐treated cultured cells or WT HeLa cells were then infected with EPEC (JPN15) (Figure 3). Although pedestals formed in cells with undetectable levels of Palladin, they were significantly smaller and still recruited F‐actin (Figure 3) (Palladin siRNA–treated cell pedestal length = 0.8578 μm vs. control siRNA–treated cell pedestals = 1.676 μm). Then, we investigated whether adding GFP–Palladin could restore pedestal length in cells that were KDs for Palladin (Figure 4). To test this, the samples were infected with EPEC (JPN15). In a similar manner to pedestals formed in WT HeLa cells (pedestal length 1.395 μm) and control siRNA–treated cells (pedestal length 1.302 μm), pedestals formed in Palladin KD cells, which were then transfected with GFP–Palladin prior to infection, resulted in a similar pedestal length (pedestal length 1.496 μm) (Figure 4).

### 2.4 Palladin Domain Mutations Alter EPEC Pedestal Length

**FIGURE 5:**
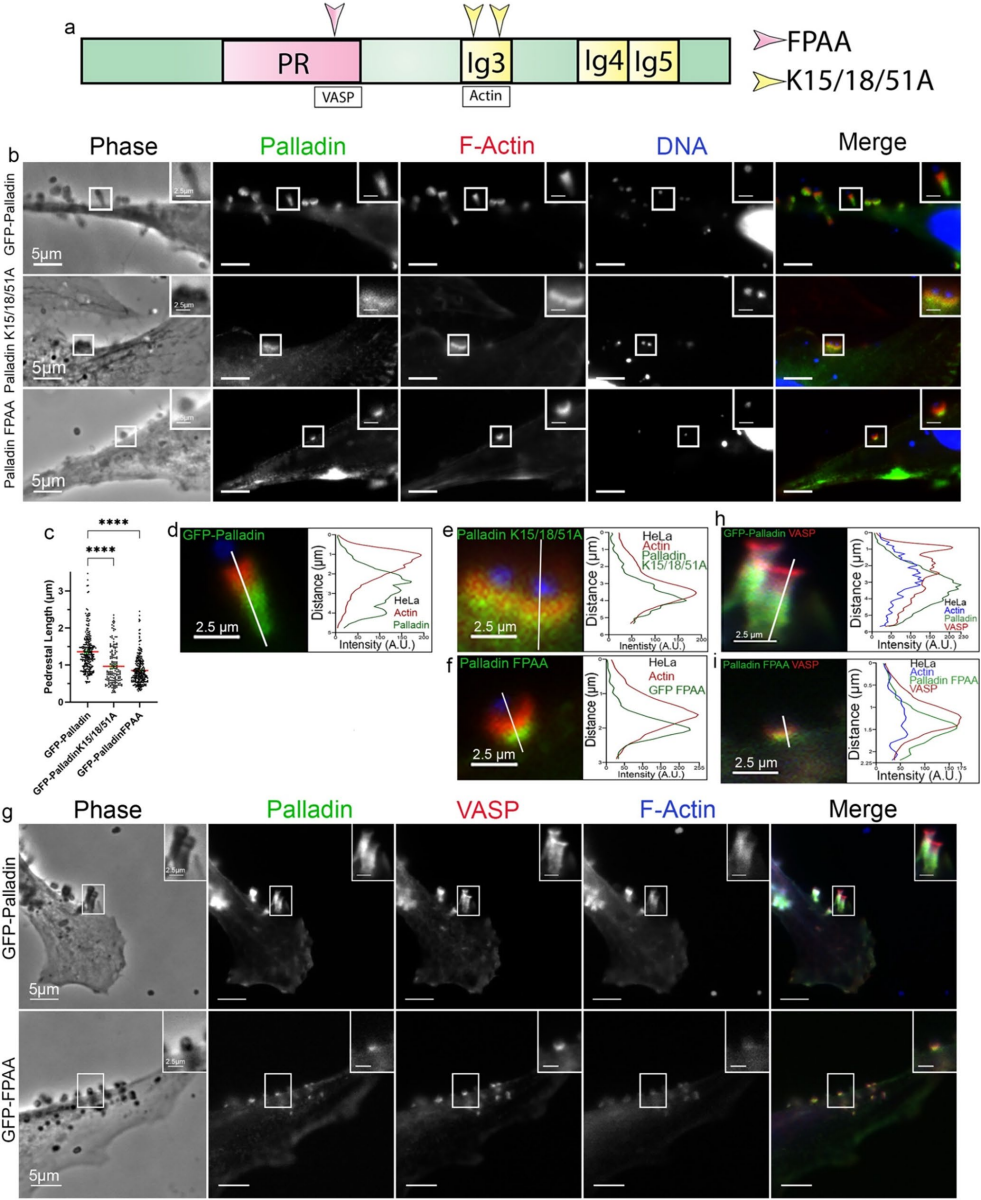
Role of Palladin domains at EPEC pedestals. (a) Diagram of Palladin showing the FPAA (pink arrowhead) and K15/18/51A (yellow arrowheads) mutation sites. (b) Transfected GFP‐Palladin, GFP‐ K15/18/51A (actin‐binding mutant), or GFP‐FPAA (VASP‐binding mutant) in HeLa cells followed by infections with JPN15 for 8 h. Cells were stained with Alexa 594‐phalloidin (red) to visualize F‐actin and DAPI (blue) to visualize host cell DNA and bacteria. The insets are enlargements of boxed regions. Scale bars are 5 or 2.5 μm (inset). (**c**) Pedestal length analysis (K15/18/51A = 0.9650 μm and FPAA = 0.8539 μm from 158 and 257 EPEC pedestals, respectively), while GFP‐Palladin had pedestals that measured 1.36 μm (from 258 EPEC pedestals). Quantification of pedestal lengths from three separate experiments (where each experiment was run in triplicate) was obtained and plotted as a scatter plot (relative to control [±SEM]). One‐way analysis of variance (ANOVA) followed by Tukey's multiple comparisons test was used to assess the significance of the EPEC pedestal length in GFP‐Palladin transfected HeLa cells compared with GFP‐K15/18/51A and GFP‐FPAA transfected HeLa cells. Asterisks indicate significant differences (*p* < 0.0001). The length of the EPEC pedestals was determined the same as previously described in Figure 3. (d–f) The white line was drawn through the EPEC pedestal to show F‐actin and palladin distribution/relative abundance. Scale bar = 2.5 μm. (g) To confirm whether or not VASP remained associated at FPAA‐mutated pedestals, we transfected in GFP‐Palladin or the VASP‐binding mutant (GFP‐FPAA), infected the cells with JPN15 for 8 h, and stained for VASP. VASP localization was retained at the shortened pedestals. (h and i) The white line was drawn through the EPEC pedestal, and the total F‐actin intensity (blue) as well as the corresponding Palladin (green) and VASP (red) were plotted. Scale bar = 2.5 μm.

The actin‐ and VASP‐binding domains of Palladin are important for its normal functioning (Beck et al. 2013; Gateva et al. 2014). To examine the importance of these regions during EPEC pedestal formation, we overexpressed Palladin domain mutants in cultured cells prior to infecting them with EPEC. Palladin's ability to bind to actin has been tied to three lysine residues (K15, K18, and K51) within its Ig3 domain (Figure 5). When we examined pedestals in the transfected cells, we found that the K15/18/51A pedestals were significantly shorter than the pedestals formed in WT‐Palladin–transfected cells (GFP‐K15/18/51A = 0.9650 μm vs. GFP‐WT = 1.360 μm), indicating that the constructs showed dominant negative effects. Similarly, pedestal length was significantly decreased when a Palladin VASP (GFP‐FPAA) domain mutant was used in place of GFP‐Palladin (GFP‐FPAA pedestals measured 0.8539 μm) (Figure 5). Interestingly, when the VASP domain of Palladin was deleted, VASP remained localized at the short pedestals (Figure 5).

### 2.5 Overexpressed Palladin Rescues EPEC Pedestal Length in ArpC2‐Depleted Cells

**FIGURE 6:**
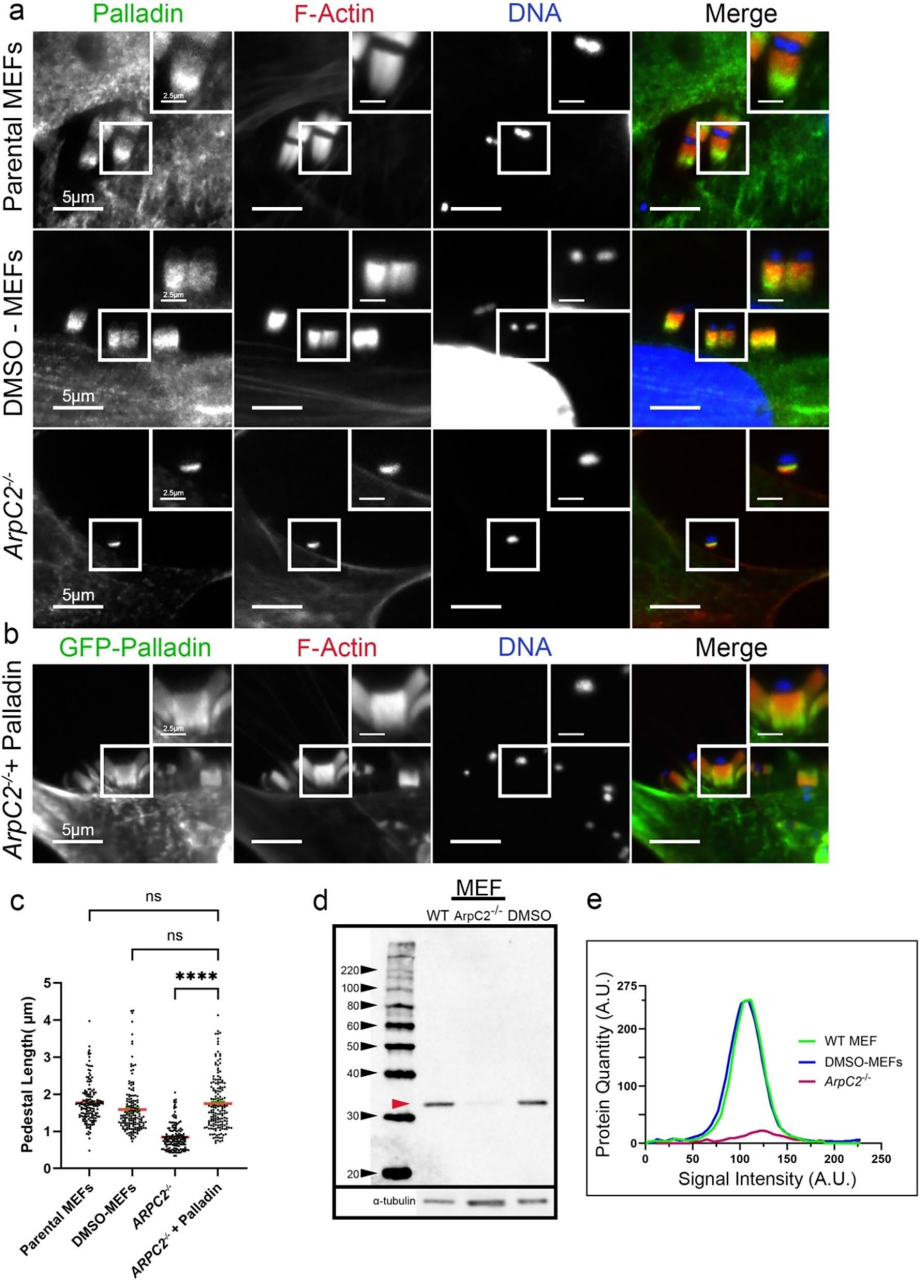
Palladin rescues pedestal length in *ArpC2* ^*−/−*^ cells. (a) Parental MEFs, DMSO controls, and *ArpC2* ^*−/−*^ MEFs stained with Palladin‐targeting antibody (green), Alexa 594‐phalloidin (red) to visualize F‐actin, and DAPI (blue) to visualize host cell DNA and bacteria. The insets are enlargements of boxed regions. Scale bars are 5 or 2.5 μm (inset). (b) Pedestal length was restored when *ArpC2* ^*−/−*^ cells overexpressed GFP‐Palladin. Cells were stained with Alexa 594‐phalloidin (red) to visualize F‐actin and DAPI (blue) to visualize host cell DNA and bacteria. Scale bars are 5 or 2.5 μm (inset). (c) Quantification of pedestal lengths from three separate experiments (where each experiment was run in triplicate) was obtained and plotted as a scatter plot (relative to control [±SEM]). One‐way analysis of variance (ANOVA) followed by Šidák multiple comparisons test was used to assess the significance of the EPEC pedestal length in parental MEFs compared with *ArpC2* ^*−/−*^+ Palladin (not significant (ns)), DMSO‐MEFs compared with *ArpC2* ^*−/−*^+ Palladin (ns), and *ArpC2* ^*−/−*^compared with *ArpC2* ^*−/−*^+ Palladin (*p* < 0.0001). Asterisks indicate significant differences. The average pedestal length of the parental MEFs was 1.767 μm (from 156 EPEC pedestals). The DMSO‐MEFs had an average pedestal length of 1.591 μm (from 156 EPEC pedestals). The average pedestal length of *ArpC2* ^*−/−*^ 0.8451 μm (from 153 EPEC pedestals) was significantly shorter than the parental‐MEFs and DMSO‐MEFs (data not shown). When GFP‐Palladin was transfected into *ArpC2* ^*−/−*^, the average pedestal length was 1.749 μm (from 161 EPEC pedestals), which was significantly longer compared to the *ArpC2* ^*−/−*^. The length of the EPEC pedestals was determined the same as previously described in Figure 3. (d) Whole MEF lysates from uninfected cells, where WT is untreated MEFs, *ArpC2* ^*−/−*^ is 4‐OHT treated MEFS, and DMSO is the control MEFs, were probed for endogenous ArpC2 using rabbit polyclonal anti‐p34/Arc/ArpC2 polyclonal antibody antibodies (indicated by a solid red arrowhead). α‐Tubulin is shown as a loading control. A slight band in the *ArpC2* ^*−/−*^ lane is evident as is the overloading of protein in that lane. (e) The graph depicts the densitometry of the protein quantity in the western blot shown in Figure 6d. It analyzes the integrated signal intensity over the protein quantity based on the pixels analyzed.

The *ArpC2* ^*−/−*^ cell line was established many years ago (Rotty et al. 2015) and uses a 4‐hydroxy‐tamoxifen (4‐OHT) treatment to induce the loss of the ArpC2 gene product (p34). This causes the depletion of additional Arp2/3 complex subunits, including Arp2 and Arp3 (Rotty et al. 2015). We found that upon depletion of ArpC2, small (0.8451 μm) actin pedestals remained beneath the areas of EPEC attachment (Figure 6). Pedestals in the parental mouse embryonic fibroblasts (MEFs) and DMSO‐treated control MEF cells generated EPEC pedestals of the expected sizes (1.767 and 1.591 μm) (Figure 6). Western blotting showed minute amounts of ArpC2 (p34) in overloaded and overexpressed wells with *ArpC2* ^*−/−*^ cell lysates (Figure 6). Palladin has the ability to cross‐link actin filaments into a network‐like pattern at the early stages of actin polymerization during pure protein assays (Gurung et al. 2016). Additionally, pure protein assays in the absence of the Arp2/3 complex have generated actin‐rich structures associated with *Listeria monocytogenes* bacteria (Dhanda et al. 2018). Consequently, we wondered whether overexpressing Palladin could rescue pedestal length in *ArpC2* ^*−/−*^ EPEC‐infected cells.

**FIGURE 7:**
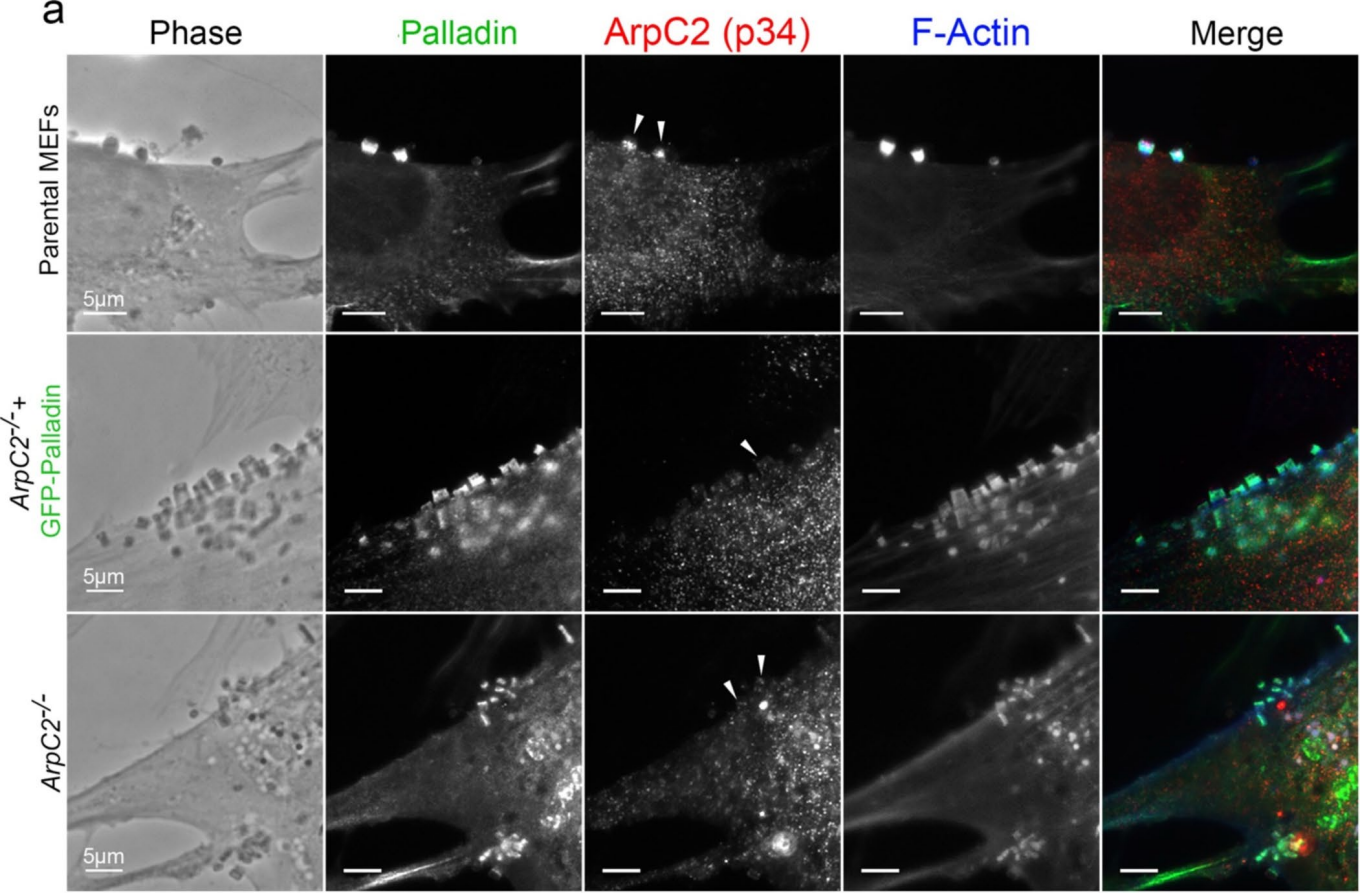
ArpC2 is not concentrated at EPEC pedestals in *ArpC2* ^*−/−*^ cells. (a) Parental MEFs, *ArpC2* ^*−/−*^ MEFs + GFP‐Palladin, and *ArpC2* ^*−/−*^ MEFs stained with Palladin‐targeting antibodies (green), ArpC2 (p34)‐targeting antibodies (red), and Alexa 405‐phalloidin (blue) to visualize F‐actin. In the *ArpC2* ^*−/−*^ MEFs + GFP‐Palladin and *ArpC2* ^*−/−*^ MEFs, ArpC2 is not concentrated at EPEC pedestals above cytosolic levels. Solid arrowheads are pointing at EPEC pedestals. Scale bar = 5 μm.

When GFP‐Palladin was transfected into the *ArpC2* ^*−/−*^ cells prior to the addition of EPEC (JPN15), pedestal length returned to wild‐type levels, resulting in pedestals with an average length of 1.749 μm, which was not significantly different from those in WT‐MEFs and DMSO control–treated MEFs, but was significantly different from those formed in the *ArpC2* ^*−/−*^ infected cells (Figure 6). In parental MEFs infected with EPEC (JPN15), ArpC2 co‐localized with Palladin midway through the pedestals. In contrast, in *ArpC2* ^*−/−*^ cells and *ArpC2* ^−/−^ cells transfected with GFP‐Palladin, any residual ArpC2 was not concentrated at the pedestals by immunolocalization (Figure 7).

### 2.6 mDia1 Within EPEC Pedestals in *ArpC2* ^*−/−*^ Cells

**FIGURE 8:**
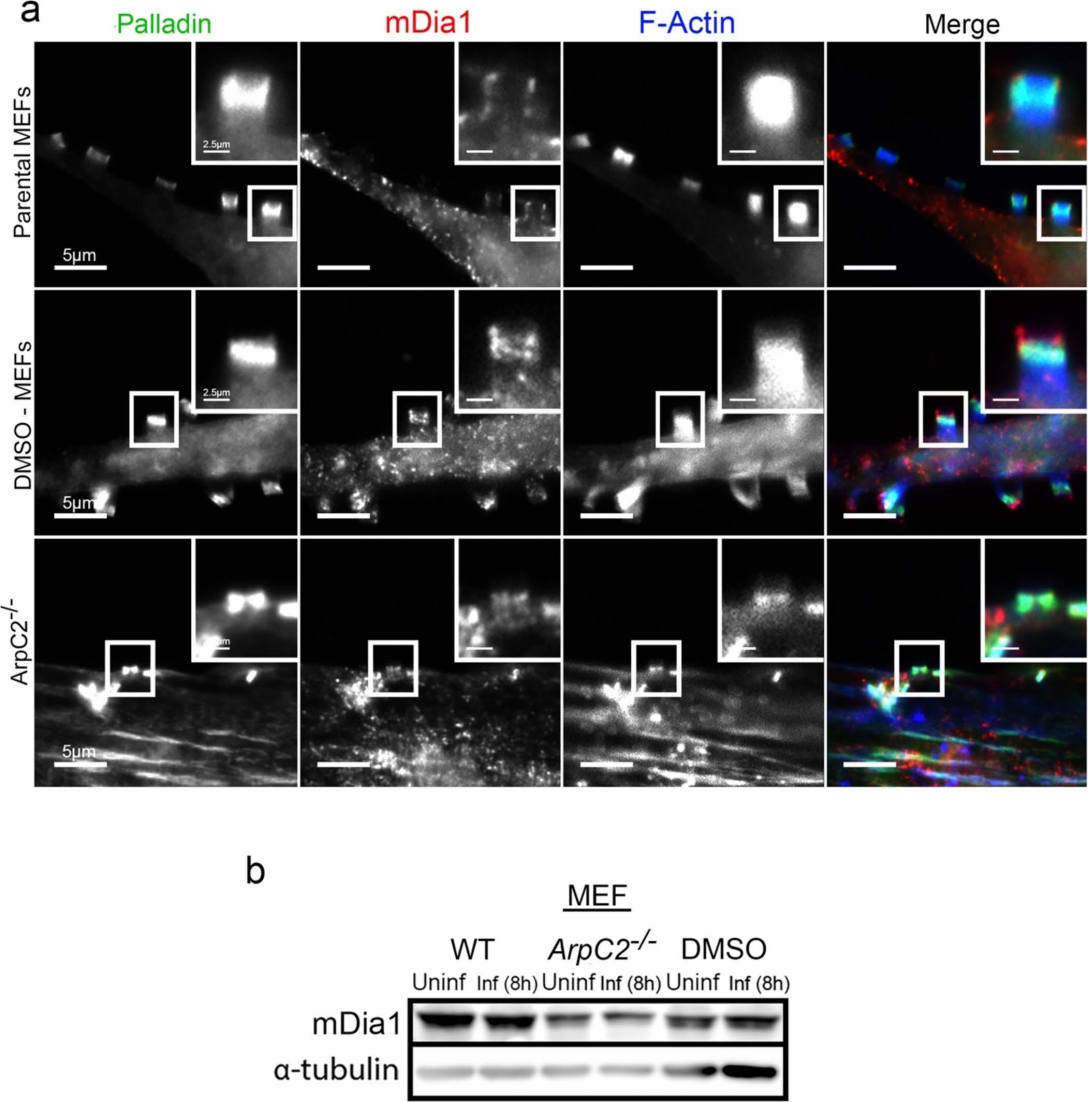
mDia1 localizes within the EPEC pedestals in *ArpC2* ^*−/−*^ cells. (a) Parental MEFs, DMSO—MEFs, and *ArpC2* ^*−/−*^ MEFs stained with Palladin‐targeting antibodies (green), mDia1‐targeting antibodies (red), and Alexa 405‐phalloidin (blue) to visualize F‐actin. Scale bars are 5 or 2.5 μm (inset). mDia1 localized as puncta within the EPEC pedestals. (b) mDia1 protein levels remain unaltered during EPEC infections. Whole MEF lysates from uninfected or JPN15 infected cells, where WT is untreated MEFs, *ArpC2* ^*−/−*^ is 4‐OHT–treated MEFs, and DMSO is the control MEFs, were probed for endogenous mDia1 using rabbit anti‐mDia1 antibodies. α‐Tubulin is shown as a loading control.

Velle and Campellone found that mDia1 localized toward the base of EPEC pedestals and that its presence was needed for efficient pedestal formation (Velle and Campellone 2018). To determine whether the shorter pedestals found in ArpC2‐depleted cells retained mDia1 in the pedestals, we infected the *ArpC2* ^*−/−*^ cells as well as the parental MEFs and DMSO‐MEF controls and immunolocalized mDia1 after 8‐h infections. In the parental MEFs and DMSO‐MEFs, we observed that mDia1 localized as puncta (but in linear arrangement) within the EPEC pedestals (Figure 8a). mDia1 levels did not noticeably change in infected cells (Figure 8b).

## 3 Discussion

Palladin is a well‐known actin‐associating protein. Consequently, we expected Palladin to colocalize perfectly with the actin at EPEC pedestals, but that was not the case. Although there was some colocalization with actin, Palladin concentrated at the pedestal base, beneath the area where actin staining was most intense within the structures. There are a handful of other proteins that also concentrate primarily in the basal regions of EPEC pedestals, namely spectrin and some of its associated proteins (Ruetz, Cornick, and Guttman 2011), as well as nexilin (Law et al. 2012) (another actin‐associated protein) and the formin mDia1 (Velle and Campellone 2018). Through the use of Palladin domain mutants, we have described important Palladin regions that influenced the length of EPEC pedestals. The overarching question is: What is Palladin doing at the base of the pedestals? Palladin's concentration in the basal regions of the pedestal could serve a stabilizing role to ensure proper length is accomplished as the pedestals extend the host cell plasma membrane. The shortening of pedestals, but not their ablation following the depletion of Palladin or the overexpression of mutant forms of Palladin, supports this.

It is well established that EPEC pedestals rely on the Arp2/3 complex for their proper formation and that the actin within the structures forms a branched network (Kalman et al. 1999; Ruetz, Vogl, and Guttman 2012). In MEFs that had an inducible knockout of ArpC2, pedestal lengths decreased substantially but were not completely absent following 8‐h EPEC infections. We postulate that, despite the absence of immunolocalized ArpC2 at pedestals, the residual ArpC2 detected after the 4‐OHT treatment (based on western blotting) could have been sufficient for actin nucleation, resulting in the observed accumulation of filamentous actin in the stunted pedestals of the *ArpC2* ^*−/−*^ cells. The nucleated actin filaments could allow Palladin to bind and exert its functions to rescue pedestal length through three potential mechanisms. First, through the association of Palladin with other actin‐associated proteins. How can this happen if endogenous Palladin concentrates at the bottom of the pedestals, not where the majority of actin is present? Previous studies have indicated that Palladin can cross‐link actin filaments (Beck et al. 2013). In addition, Palladin binds to a number of actin‐regulating proteins that all play a role in EPEC pedestal formation, including VASP (Boukhelifa et al. 2004), profilin (Boukhelifa et al. 2006), α‐actinin (Rönty et al. 2004), Ezrin (Mykkänen et al. 2001), and signalling intermediaries like Src (Rönty et al. 2007). Perhaps, the overexpression of Palladin, along with the recruitment of actin‐regulating proteins, could lead to the elongation of EPEC pedestals to WT lengths. The second potential mechanism is by branching actin filaments. Palladin has been shown to form branched actin filament structures in vitro (Dhanda et al. 2018; Gurung et al. 2016). Consequently, the nucleated actin filaments that we saw in the short pedestals formed in the EPEC‐infected *ArpC2* ^*−/−*^ cells could be a result of residual functioning Arp2/3 complex, which acts as a starting point for Palladin to bind for subsequent filament branching. Third, a previous study by Velle and Campellone (2018) showed that the formin, mDia1, contributes to both the generation and maintenance of Arp2/3‐dependent actin polymerization at EPEC pedestals. They hypothesized that mDia1 could provide short linear filaments to the Arp2/3 complex to produce the branched actin network observed in EPEC pedestals. Consequently, it is possible that Palladin could bind to the mDia‐associated linear‐actin filaments (just as Palladin does when it binds to linear actin within stress fibres) (Rönty et al. 2004). Once the Arp2/3 complex is depleted, so that only enough is present to initiate actin nucleation of the filaments, Palladin could function to branch those filaments.

## 4 Methods and Materials

### 4.1 Cell Culture

Human cervical (HeLa) cells were obtained from the American Type Culture Collection (ATCC) (catalogue no. CCL‐2). HeLa cells were cultured in Dulbecco's modified Eagle's medium (DMEM) containing high levels of glucose (HyClone; GE Healthcare) and supplemented with 10% fetal bovine serum (FBS) (Gibco, Thermo Fisher Scientific). The MEF cell line *ArpC2* ^−/−^ was generated previously by Rotty et al. (2015). Those cells were treated with puromycin dihydrochloride (Sigma) and 4‐OHT (Sigma) to obtain *ArpC2* ^−/−^ cells, while the control cells were treated with DMSO as described previously (Rotty et al. 2015). The cell lines were maintained in a cell culture incubator (37°C, 5% CO_2_). To seed cells for experiments, cells were washed 3× with Dulbecco's phosphate‐buffered saline without Ca^2+^ and Mg^2+^ (PBS[−/−]) (Gibco, Thermo Fisher Scientific), trypsinized with 0.05% trypsin–EDTA (Gibco, Thermo Fisher Scientific), and seeded onto clear polystyrene 6‐ or 24‐well plates containing glass coverslips.

### 4.2 Bacterial Strains and Growth Conditions

EPEC strainE2348/69 (Taylor 1970), EPEC ∆ *escN* (Gauthier, Puente, and Finlay 2003), EPEC (strain JPN15) (Knutton et al. 1997), and mutants from the same strain including ∆ *tir* (DeVinney et al. 2001), ∆ *tir+tir* (DeVinney et al. 2001), and ∆t*ir*+tirY474 (DeVinney et al. 2001) were used throughout this study and were grown at 37°C in Luria‐Bertani (LB) agar or LB broth (BD Biosciences) at 37°C, standing, for 12–16 h.

### 4.3 Bacterial Infections

To infect HeLa cells, broth cultures of EPEC (incubated overnight) were diluted 10‐fold (final volume of 100 μL) with prewarmed DMEM containing FBS (37°C). Four microliters (6‐well culture plate) or 2 μL (24‐well culture plate) of diluted bacteria were added into culture plates containing host cells with fresh DMEM + 10% FBS, then incubated for 8 h at 37°C to study pedestal formation. Prior to the infections, MEFs were seeded into three separate six‐well plates. The following day, the MEFs either had a fresh media change or were treated with media containing DMSO or 4‐OHT. The cells were treated every other day for a total of 5 days. On the fifth day, the cells were seeded into six‐well culture plates with the prospective media. The following day, the cells were infected, as described above. When cells were transfected with GFP‐Palladin, they were seeded on the fifth day, transfected on the sixth day, and infected on the seventh day, as described above.

### 4.4 Antibodies and Reagents

The antibodies used in this study included the following: mouse anti‐Palladin antibody (500 μg/mL for western blotting) from Novus Biologicals Inc.; rabbit CoraLite Plus 488–conjugated Palladin polyclonal antibody (0.6 μg/mL for immunofluorescence) (Proteintech); rabbit anti‐p34 antibody (2 μg/mL for immunofluorescence [biotechne]); rabbit anti‐mDia1 polyclonal antibody (2.07 μg/mL for immunofluorescence, 8 μg/mL for western blotting [proteintech]; rabbit anti‐VASP antibody) (1.1 μg/mL for immunofluorescence [Millipore]); Alexa Fluor 594–conjugated goat anti‐rabbit antibody (2 μg/mL [Invitrogen]); rabbit α‐tubulin polyclonal antibody (2.52 μg/mL [Invitrogen]) for western blotting; mouse anti‐α‐tubulin (1:500 for western blotting (Developmental Studies Hybridoma Bank [DSHB]), 12G10); rabbit‐anti‐p34/Arc/ArpC2 polyclonal antibody (MilliporeSigma) (10 μg/mL) for Western blotting; and horseradish peroxidase (HRP)–conjugated goat anti‐mouse or anti‐rabbit antibodies (1 μg/mL) (Invitrogen). The reagents used in this study included the following: Alexa Fluor 594–conjugated phalloidin, Alexa Fluor 405–conjugated phalloidin, and Alexa Fluor 350–conjugated phalloidin (Invitrogen).

### 4.5 Immunolocalization

For immunofluorescence studies, cells previously transfected with a GFP‐DNA construct or destined for treatment with anti‐VASP antibodies were fixed at room temperature using prewarmed (37°C) 3% paraformaldehyde for 15 min (prepared in 150 mM NaCl, 4 mM Na/KPO_4_, 5 mM KCl [pH 7.3], 100 mM NaOH) and then washed 3× with PBS [−/−] for 10 min. These cells were permeabilized with room temperature 0.1% Triton X‐100 (prepared in PBS [−/−]) for 5 min and then washed 3× with PBS [−/−] for 10 min. Samples exposed to anti‐VASP antibody were first blocked in 5% normal goat serum in TPBS/BSA (0.05% Tween‐20 and 0.1% BSA in PBS) for 20 min. Then, the antibody was prepared in TPBS/BSA and incubated overnight. The next day, coverslips were washed 3× with TPBS/BSA for 10 min and then treated with secondary antibodies (Alexa Fluor 594–conjugated goat anti‐rabbit; prepared in TPBS/BSA) at room temperature (in the dark) for 2 h. Following this, cells were washed 3× with TPBS/BSA for 10 min and then incubated with Alexa Fluor 350–conjugated phalloidin (prepared in TPBS/BSA) at 37°C for 1 h to visualize F‐actin. Samples were washed 3× with TPBS/BSA for 10 min and mounted onto glass microscope slides using ProLong Glass Antifade Mountant.

Cells destined for treatment with CoraLite Plus 488–conjugated Palladin polyclonal antibody, anti‐p34, or anti‐mDia1 were also fixed with paraformaldehyde as stated above, but permeabilized with chilled acetone (−20°C) for 7 min, then air dried at room temperature for 30 min. Following this, samples with GFP‐DNA constructs were incubated with Alexa Fluor 594–conjugated phalloidin (prepared in PBS [−/−]) for 15 min to visualize F‐actin. Samples were washed 3× with PBS [−/−] for 10 min and mounted onto glass microscope slides using Prolong Diamond antifade mountant containing DAPI (4′,6‐diamidino‐2‐phenylindole; Invitrogen). Samples exposed to the rabbit CoraLite Plus 488–conjugated Palladin polyclonal antibody were first blocked in 5% normal goat serum in TPBS/BSA for 20 min. Then, the antibody was prepared with TPBS/BSA and incubated on the coverslips at 37°C for 1 h. Afterward, the coverslips were washed 3× with TPBS/BSA. After the final wash, the coverslips were exposed to Alexa Fluor 594–conjugated phalloidin (prepared in TPBS/BSA for 15 min), which was followed by washing 3× with TPBS/BSA for 10 min and mounted onto glass microscope slides using Prolong Diamond antifade mountant containing DAPI (4′,6‐diamidino‐2‐phenylindole; Invitrogen).

MEF cells destined for staining with anti‐p34 or anti‐mDia1 antibodies (together with GFP‐Palladin) were first blocked in 5% normal goat serum in TPBS/BSA for 20 min. Then, they were exposed overnight to either the anti‐p34 or anti‐mDia1 polyclonal antibodies prepared with TPBS/BSA. The following day, the samples were washed 3× with TPBS/BSA for 10 min and then treated with secondary antibodies (Alexa Fluor 594–conjugated goat anti‐rabbit) at room temperature in the dark for 2 h. Then, the samples were washed 5× with TPBS/BSA, with 10 min incubation for the last wash, and repeated 3×. Following these washes, the samples were exposed to the rabbit CoraLite Plus 488–conjugated Palladin polyclonal prepared with TPBS/BSA and incubated on the coverslips at 37°C for 1 h. Afterward, the coverslips were washed 3× with TPBS/BSA. After the final wash, the coverslips were exposed to Alexa Fluor 405–conjugated phalloidin (prepared in TPBS/BSA) for 1 h at 37°C. This was followed by washing 3× with TPBS/BSA for 10 min and mounting onto glass microscope slides using ProLong Glass Antifade Mountant.

### 4.6 Lysate Preparation and Western Blotting

Lysates were prepared by washing the samples 3× with prewarmed PBS [−/−] and then the samples were treated with ice‐chilled sterile radioimmunoprecipitation assay (RIPA) lysis buffer (150 mM NaCl, 50 mM Tris [pH 7.4], 5 mM EDTA, 1% Nonidet P‐40, 1% deoxycholic acid, 10% SDS) containing the cOmplete Mini EDTA‐free protease inhibitor cocktail (Roche) for 10 min. Using cell scrapers, cells were disrupted, and lysates were collected into microcentrifuge tubes. Lysates were centrifuged at 4°C and 16,278×*g* for 10 min to pellet cellular debris. To determine protein concentrations, a bicinchoninic acid (BCA) assay kit (Pierce) was used. For Western blotting, lysate samples were first prepared in 6× SDS protein–loading buffer and then boiled for 10 min. Equal amounts of protein were loaded onto 10% SDS–polyacrylamide gels and resolved by electrophoresis. Following separation, gels were transferred onto nitrocellulose membranes using a Trans‐Blot SD semidry transfer cell (Bio‐Rad). After the transfer, the membranes were washed for 10 min in TBST (Tris‐buffered saline, 0.05% Tween 20) with shaking, and blocked with 4% Blotto (Santa Cruz Biotechnology) prepared in TBST for 1 h shaking, then treated with primary antibodies (diluted in TBST plus 1% BSA) overnight at 4°C. The membranes were then washed for 30 min prior to incubation with secondary antibodies (HRP‐conjugated goat anti‐mouse or anti‐rabbit antibodies) for 1 h at room temperature with shaking. Following the manufacturer's instructions, the membranes were treated with Western Lightning Plus‐ECL (PerkinElmer) and visualized using an AI 600 RGB imager. Using a mild stripping buffer (1.5% glycine, 0.1% SDS, 1% Tween 20 [pH 2.2]), membranes were stripped of bound antibodies to confirm equal loading and re‐probed using rabbit or mouse anti–α‐tubulin–targeting antibody as outlined above.

### 4.7 DNA Constructs

The Palladin constructs used consisted of the 90 kDa isoform (Beck et al. 2013). These vectors included GFP vectors containing wild‐type Palladin and the actin‐binding mutant GFP‐PalladinK15/18/51A previously made by Beck et al. (2013) and GFP‐PalladinFPAA (Dhanda et al. 2018).

### 4.8 Transfections

All DNA transfections of cultured cells were performed using jetPRIME transfection reagents (Polyplus Transfection) according to the manufacturer's instructions. Briefly, cells were transfected (3 μL reagent and 1.5 μg plasmid DNA per six‐well, or 400 ng plasmid DNA per 24‐well) and allowed to incubate at 37°C for 4 h. Following this, the medium was replaced, and cells were incubated for 24 h at 37°C to allow the expression of the respective gene product. Transfected cells were fixed for viewing by immunofluorescence microscopy.

### 4.9 Transfection of siRNA

A smart pool (ON‐Targetplus siRNA) of four Palladin siRNAs and four non‐targeting control siRNA was purchased from Dharmacon (GE Healthcare). Following the manufacturer's instructions, the transfections were performed using the siRNA transfection reagent INTERFERin (Polyplus transfection). Seventy‐two hours after siRNA treatment, cells had decreased levels of Palladin protein. For Figure 4, the siRNA treatments lasted for 48 h, during which media were changed for all siRNA‐treated cells. Additionally, a population of Palladin siRNA–treated cells were transfected with GFP‐Palladin, as discussed below.

### 4.10 Microscopy

Images were acquired using a Leica DMI4000B (Leica Microsystems Inc.) inverted fluorescence microscope and a Hamamatsu Orca R2 charge‐coupled device (CCD) camera (Hamamatsu Photonics). All devices were controlled by MetaMorph Imaging System software (Universal Imaging). Images were also obtained through this software. Adobe Photoshop 2022 was used to process the acquired images for figures. Fiji was used to analyze Figure 6D to create Figure 6E.

### 4.11 Statistical Analysis

Statistical analysis was performed using GraphPad Prism version 10.2.1. K.M.B. performed the experiments, along with the enumeration and statistical analyses. All data were obtained from experiments performed at least three times (*n* = 3). The presented microscopy images are representative of experiments conducted.

## Ethics Statement

The authors have nothing to report.

## Consent

I, Kaitlin M. Bruzzini, Julian A. Guttman, and S. Tara Mann, consent for publication.

## Conflicts of Interest

The authors declare no conflicts of interest.

## Data Availability

Raw Data Available Upon Request to Julian A. Guttman.
